# Differentiating innovation priorities among stakeholder in hospital care

**DOI:** 10.1186/1472-6947-13-91

**Published:** 2013-08-16

**Authors:** Mattijs S Lambooij, Marjan J Hummel

**Affiliations:** 1 Centre for Prevention and Health Services Research, National institute of Public Health and the Environment (RIVM), P.O. Box 1, 3720, BA Bilthoven, The Netherlands; 2 Department of Health Technology & Services Research, MIRA, University of Twente, P.O. Box 217, 7500, AE Enschede, The Netherlands

**Keywords:** Implementation, Information technology, Innovation, Hospital care, Stakeholders

## Abstract

**Background:**

Decisions to adopt a particular innovation may vary between stakeholders because individual stakeholders may disagree on the costs and benefits involved. This may translate to disagreement between stakeholders on priorities in the implementation process, possibly explaining the slow diffusion of innovations in health care. In this study, we explore the differences in stakeholder preferences for innovations, and quantify the difference in stakeholder priorities regarding costs and benefits.

**Methods:**

The decision support technique called the analytic hierarchy process was used to quantify the preferences of stakeholders for nine information technology (IT) innovations in hospital care. The selection of the innovations was based on a literature review and expert judgments. Decision criteria related to the costs and benefits of the innovations were defined. These criteria were improvement in efficiency, health gains, satisfaction with care process, and investments required. Stakeholders judged the importance of the decision criteria and subsequently prioritized the selected IT innovations according to their expectations of how well the innovations would perform for these decision criteria.

**Results:**

The stakeholder groups (patients, nurses, physicians, managers, health care insurers, and policy makers) had different preference structures for the innovations selected. For instance, self-tests were one of the innovations most preferred by health care insurers and managers, owing to their expected positive impacts on efficiency and health gains. However, physicians, nurses and patients strongly doubted the health gains of self-tests, and accordingly ranked self-tests as the least-preferred innovation.

**Conclusions:**

The various stakeholder groups had different expectations of the value of the nine IT innovations. The differences are likely due to perceived stakeholder benefits of each innovation, and less to the costs to individual stakeholder groups. This study provides a first exploratory quantitative insight into stakeholder positions concerning innovation in health care, and presents a novel way to study differences in stakeholder preferences. The results may be taken into account by decision makers involved in the implementation of innovations.

## Background

Technological innovations in health care have cured diseases, reduced harm and risk in surgical procedures, prolonged the average life expectancy and consequently increased demand for additional care with corresponding costs [1,2]. In many countries, policy makers and management believe that innovation, and especially the uptake of information technology (IT) innovations, will make a major contribution to improved efficiency in health care [3]. Adoption of IT-innovations is supposed to create sustainable health care through increasing the efficiency of care processes and increasing the retention and attraction of employees by providing challenging and meaningful work.

However, contrary to these beliefs and despite an abundance of novel ideas and work practices, the implementation of innovations in hospitals is hampered [4]; 10,000 new studies per year are published in MEDLINE, but many of these potentially beneficial IT-based [5-7] innovations fail to reach the target groups and are not applied in daily practice. This failing may be understood by addressing the questions of who benefits from which innovation and who bears the costs. There are numerous examples of innovations implemented in health care that in hindsight failed to deliver obvious benefits [8,9], or for which the perceptions of the benefits vary between the actors involved [10].

An innovation is a practice or object that is perceived as new by the actor who adopts it [11]. We assume that an actor will adopt (start to use or implement) an innovation when the perceived benefits of using the innovation outweigh the perceived costs, and hence, the new practice or object is an improvement on the current situation. Grol et al. [4] summarized the factors that affect the success of implementing change (including innovations) in health care: features of the innovation itself, features of the target group, features of the patients, features of the social setting, financial features, administrative and organizational context, and features of the methods and strategies for dissemination and implementation used [4]. Successful technological diffusion of innovations is seen as the result of a process of mutual adaptation among technology producers, users, and external groups [11,12], and the system that adopts the innovation. The interplay of these factors can shape the properties of the new technology, the use of the technology, the organizational context, and the societal context.

Studies identify physicians as a barrier to be overcome in the implementation of, especially administrative, IT innovations [13]. However, not all delay can be blamed on physicians since some of the IT software has noteworthy flaws, adding unnecessary costs to the adoption of innovation for the users [14]. This may explain part of the users’ resistance or avoidance strategies [15,16]. These resistance or avoidance strategies may be inspired by political considerations and power struggles, which may affect the diffusion of innovations as much as more ’rational’ considerations such as benefits of the innovation [17].

In this paper, we focus on one aspect of the social setting of adoption of innovations: stakeholders’ preferences regarding IT innovations and their expected benefits. Monetary costs may be an important cause for slow diffusion [18]. In this study, however, the costs and benefits are broader than monetary. The costs to and benefits for the different actors will be at different levels of the innovation process: the environmental level (e.g., a benefit may be improved exchange of information; a cost may be renewing work flow with external partners); the organizational level (e.g., a benefit may be solving an organizational problem; a cost may be investment in new infrastructure, resistance in the organization, providing employees with new skills (training, education)); the social level (social support or negative attitudes of co-workers); the psychological level (feelings of improved self-efficacy or loss of control); and the user level (better quality of work, versus initially being less productive because a new way to work has to be learned) [19,20].

Stakeholders are known to disagree on the relative importance of innovations and may therefore use their resources to influence other stakeholders [21] and resort to politics and power to affect implementation processes. Politics and the power balance between stakeholders may be particularly important for innovations that span a large part of an (health care) organization where multiple stakeholders holding different positions in the organization are mutually dependent in the implementation and utilization of the innovation. The different positions of stakeholders and the concomitant differences in priorities and agendas are likely to affect each stage of the implementation process. In all stages, from the first stage of experiencing and defining a problem, to looking for solutions, to balancing investments against the improvements and the evaluation, the differences in structural (stakeholder) positions will affect the costs and benefits for the different stakeholders and the consequent preferences and priorities of the stakeholders.

The understanding of differences in positions between stakeholders thwarting the implementation of innovations is not new but has received little attention in empirical research so far, as far as we know [11,22-24]. It has been argued that when the implementation of clinical information systems is purely seen as a technical implementation rather than a complex social implementation, the chances of failure are larger [16,25]. In some models, the presence of stakeholders is mentioned [17], and studies have shown that when stakeholders disagree on the priority of the innovation, uptake will be difficult [26]. Applying stakeholder theory [27] to the implementation of innovations in health care, organizations may better understand why the diffusion of possibly valuable innovations proceeds more slowly than many stakeholders wish it would. Because of this slower diffusion, the potential benefits of IT innovations may be hampered unnecessarily.

The preference structures of the stakeholders are likely to be based on their perceived costs and benefits of the innovation. For instance, doctors misspelling diagnoses [15] did not do so because they wanted to be rebels, but because their perceived costs of the time lost by clicking on pop-ups were higher than the added safety value of the warning of dangerous combinations of medicines. We expect to find similar cost–benefit estimations for other innovations and for other stakeholders, explaining their respective preference structures. In turn, we expect that when the differences between these preference structures become larger, the speed of diffusion of innovations will slow down.

The questions addressed by this paper are therefore: Do different stakeholders have different preference structures concerning the relative relevance of IT-innovations in health care? Can these differences be explained by differences in the costs and benefits associated with the innovations for the stakeholders?

## Methods

### Identifying the stakeholders

Using the three decision systems put forward by Greer [28], we will first identify three groups of stakeholders involved in the implementation of innovations in hospitals: physicians and nurses (from the medical-individualistic decision system), hospital management (from the fiscal managerial decision system) and the hospital boards (from the strategic-institutional decision system). We will separate the nurses from the physicians because they hold different organizational positions within hospitals, which may affect their preference structures.

Subsequently, we take the stakeholder perspective one step further than Greer does by following Freeman, who states that stakeholders are those groups and individuals who can affect or are affected by the achievement of the organization [27]. The main stakeholder group, affected by innovations and therefore included in the study, is the patients. Two relevant stakeholders we included in the analysis, and are labeled ‘external determinants’ of the innovation decisions by Greer, are the government and the insurer. We will also include these groups in the analysis. One goal of government is to make the health care system future-proof by facilitating the diffusion of efficiency-enhancing innovations. Insurers will have to pay for many of the innovations that are introduced and will therefore also influence the process by their decisions to reimburse innovations or not.

### Selection of innovations

#### Literature search

In a two-stage selection process, we identified IT innovations in health care that may realistically be expected to be beneficial to hospital care in the (near) future. The first step was a literature scan of published research articles in Scopus and Web of Science. The queries were focused on innovations or new technologies in hospital care (Additional file 1 for search queries). This yielded 450 hits (Scopus 280, and Web of Science 119). The two authors identified the relevant articles and reached consensus on which papers to include, resulting in 140 included articles. Subsequently, the article abstracts were studied for relevant IT innovations. Fifty-one IT-innovations related to hospital care were identified. These innovations were clustered according to two dimensions: distance to the health care process (i.e., the distance depends on the frequency of interactions with patients or healthcare providers, with a high frequency implying a short distance and a low frequency implying a long distance) and size of investments (including all costs such as money, time delay, reorganization of care processes, and training, from the perspective of the stakeholder that needs to make the investment) (see Table 1).

**Table 1:** Results of a literature search, by investment and distance to a care process, for expert opinion on relevance (quality, efficiency and sustainability of hospital care)

	**Size of investments**		
	**Large investments in time, money and human capital strategic**	**Medium investments**	**Small investments in time, money and human capital, strategic**
**Large distance to patient care***(concerning hospital and its surroundings financial functions, analytics (not patient level))*	• Beacon community program to improve nation’s health care	• (2) Infection control knowledge management	• (1) Full-text search engine for using narrative data in electronic health records
		• (2) Antiviral information management system	
	• (2) National-level connectivity Electronic Patient File (EPF)	• Biased sample hospital-based area disease estimation	
		• Electronic systems to collect client-level vaccination data	• Performance improvement systems
			• (3) Performance management systems
			• Visual diagrammatic language techniques to analyze work processes
			• Electronic logistics information systems
			• (2) Programming model to support operating room planning
			• Administrative IT
**Medium distance to patient care***(concerning the professional and the hospital organization)*	• (3) Interorganisational Electronic Health Record (Regional)	• eReferrals to specialists	• (1) Electronic prescriptions
		• Internet and electronic data mining in pathology	• PDAs
	• Picture archiving and communication system	• Communication systems in radiology	• (1) Blackberry as a clinical communication tool
	• Emergency medicine information systems	• Electronic medical record system of outpatient workflow	• Voice recognition dictation
	• (1) Clinical information systems (e.g., in radiology)	• Identification device technology to analyze work processes	• (2) Web-based distance learning
	• (2) Patient data management system	• Computerized display of laboratory and radiology results	• Computerized ordering systems
	• (2) Electronic patient records (third generation; registration and structured documentation, clinical support), within one institution	• Systems to analyze patient waiting and progress	• (3) Tablet as a clinical information tool
		• Ambulatory referral management system (management of patient routes)	• (2) Computerized clinical decision support by desktop/laptop
			• (1) Inter-colleague consultation
		• Indoor positioning	
		• Remote-controlled transfusion management	
		• Automated medication-use process for prescribing and dispensing medication (including computerized prescriber order entry)	
		• Electronic discharge systems	
		• (3) Bar code medication administration technology	
		• Computerized physician order entry for prescribing medication and recording medication administration	
		• (2) Electronic health records within hospital	
**Small distance to patient care***(concerning interaction between the patient and care professional)*	• (1) Planning software surgery	• (1) Remote patient management	• (4) Virtual consultations
	• (2) Computer-assisted surgery, using a robot	• (3) Telenursing (application of Telecare)	• (2)Voice link between elderly and caregivers (part of assisted living facility)
	• (1) Three-dimensional ultrasonography	• (4) Telepathology (application of Telecare)	
		• (3) Teleradiology (application of Telecare)	• (2) Patient safety alert system
		• (1) Smart Health Communities	• (1) Computerized clinical decision support by app on a PDA
		• (5) Self-testing and online automated management	• (2) E-triage
		• (1) Telemonitoring of heart failure (application of Telecare)	
		• Virtual microscopy system	
		• (1) Digital imaging	
		• (5) Portal by hospital for appointments and reminders	
		• (4) Portal by hospital for online consulting (mail, tweet, chat)	
		• (4) Portal by hospital for peer-group-contact patients	

These two dimensions are likely to affect the costs and benefits of different innovations for the actors involved. The size of the investment is directly tied to costs (both monetary and non-monetary aspects such as organizational change), and distance to health care may explain differences in impact on work practices for different actors. For example, in the upper right cell of Table 1, the “Full-text search engine for using narrative data in electronic health records” is likely to be beneficial to people analyzing patient data, such as analysts and decision makers, but it is unlikely that many clinicians are affected by this innovation. In the same column in the bottom row, we find “Computerized clinical decision support by app on a PDA” (a PDA is a personal digital assistant). It is likely that this innovation will affect the work practice of clinicians because of the direct availability of information to support decisions they need to make several times per day, while this innovation is not likely to majorly affect the work practice of board members (provided they are not active clinicians as well). Using these two dimensions, the innovations were divided into nine clusters (Table 1).

We asked 10 experts (see the next section for further explanation) whether they knew of potentially beneficial innovations that were not included in the table. This resulted in an additional 11 innovations, resulting in 62 innovations in the matrix. Table 1 presents the final list of innovations and how many times these innovations were selected by the experts.

#### Expert judgments in interviews

With the ensuing matrix, we approached 10 experts asking them to identify one innovation per cluster (each cell in Table 1 presented a cluster) from which the experts expected it would yield considerable progress in terms of quality of care and cost-efficient care. We allowed the experts to pick two or three innovations if they were unable to identify the innovation that was most beneficial in the specific cluster. Each time that an expert selected an innovation, the innovation was marked with a score 1. From the sum scores of their choices and their complementary justification, we selected nine innovations for the analytic hierarchy process (AHP) in the next phase.

The group of experts consisted of two active hospital physicians, one physician working outside of the hospital, one member of hospital staff, two directors of advice organizations specialized in hospital IT and management, and four researchers with expertise of IT in health care (e.g., E-health). In Table 1, the number of times each innovation was selected by the experts is given in parentheses.

From the expert scores, the following IT-based innovations were selected to be included in the AHP: regional EPF (Electronic Patient File), computer-assisted telesurgery using a robot, barcode medication administration technology, telepathology, self-testing and online automated management, a digital hospital portal, planning software to support operating room planning, a PDA with decision support, and virtual consultations (see Additional file 2 for a short explanation of and additional literature on these innovations).

### Analytic hierarchy process

We employed the AHP method as an analytical tool with which to prioritize decision alternatives using multiple criteria to quantify the differences in stakeholder positions on the selected IT innovations [29-32]. The first step of the AHP is to compose a hierarchical structure, including the objective, the main and sub-decision criteria and the alternatives. In the second step, the AHP offers a pairwise comparison approach to calculate the relative weights for each of the decision criteria and the preference priorities for the alternative innovations.

To calculate the weights, the respondents judged each pair of decision criteria on a scale from 9 to 1 to 9, with the two extremes (score 9) indicating that the present criterion is extremely more important than the one it is compared to, and the middle of the scale (1) indicating equal importance. In a similar manner, the preferences for the innovations can be judged in comparison with current care. For these pairwise comparisons, a score of 9 indicates an extreme preference for either the innovation or current care.

An average of the individual judgments in the pairwise comparisons can be calculated to represent the judgment of a group as a whole. The AHP provides a measure of consistency, showing whether each pairwise comparison is logically sound with regard to the remainder of the comparisons. When inconsistency is acceptable, weighting factors and performance priorities are calculated employing the principal right eigenvector [33]. As such, the weighting factors assigned to the outcome measures represent the relative importance of each of these outcome measures, while the priorities represent the relative preferences for the innovations. The overall priority of an innovation per stakeholder group is the weighted sum of the relative preferences for the innovation according to each decision criterion. In order to weight these relative preferences, they are multiplied by the importance of the corresponding decision criterion. See Saaty [33] for a more in-depth explanation of AHP mathematical approaches.

#### Criteria

**Figure 1:**
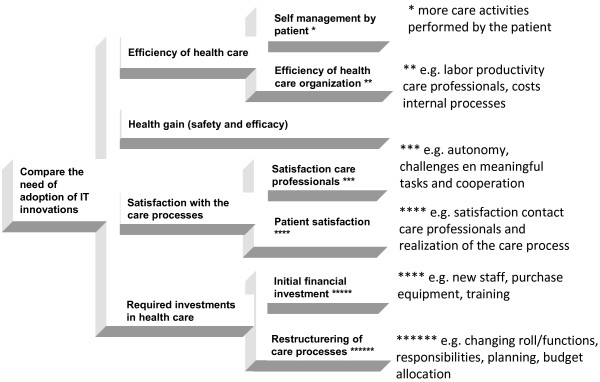
Hierarchical structure with decision criteria.

The criteria along which the innovations are judged are related to the costs and benefits on the various levels on which the stakeholders operate. The decision criteria selected are a) improvement in efficiency in health care (through 1) improvement of work processes and 2) increased self-management of patients), b) health gains of the patient after implementation of the innovation, c) satisfaction with the care process (through 1) employee satisfaction and 2) patient satisfaction), and d) required investments to implement the innovation (see Figure 1).

#### Respondents

We met the threshold of five members for all stakeholder groups, by adding one board member to the managers in the sample. We approached possible respondents through networks of the researchers and the networks of colleagues, and tried to ‘snowball’ the contacts of our respondents. The patients and nurses were gathered from a survey panel of an external research agency. Both were random samples. The nurses were included if they were working in a hospital and were involved in patient care at the moment of filling out the questionnaire. Patients were included who had been hospitalized for at least one night in the two years prior to filling out the questionnaire. Physicians were contacted through personal networks of coworkers of the authors. We included physicians that were actively working in a hospital. We succeeded in including physicians of various age groups. Managers were recruited through the networks of co-workers; we contacted three insurer companies, and at one of the companies, a number of employees working in the department involved with innovation in health care agreed to cooperate. The policymakers were recruited at the Ministry of Health, Welfare and Sport by one of the authors who spent one day a week doing several research activities. Eventually, 62 respondents were included in our sample. All respondents worked in the Netherlands at the time of filling out the questionnaire. Table 2 presents the numbers per stakeholder group in parentheses.

**Table 2:** Weighting factors of the decision criteria

**Decision criterion**	**Patients (15)**	**Nurses (22)**	**Physicians (9)**	**Managers (9)**	**Health insurers (5)**	**Policy makers (6)**
**Efficiency**	**0.22**	**0.21**	**0.20**	**0.29**	**0.29**	**0.46**
**Patient self-management**	0.45	0.35	0.28	0.24	0.45	0.64
**Efficiency of organization**	0.55	0.65	0.72	0.76	0.55	0.36
**Health gain**	**0.42**	**0.35**	**0.36**	**0.28**	**0.43**	**0.28**
**Satisfaction**	**0.28**	**0.31**	**0.33**	**0.33**	**0.18**	**0.15**
**Health care professionals**	0.21	0.52	0.42	0.26	0.21	0.26
**Patients**	0.79	0.48	0.58	0.75	0.79	0.74
**Investments**	**0.09**	**0.14**	**0.11**	**0.11**	**0.10**	**0.11**
**Initial**	0.25	0.45	0.32	0.43	0.25	0.24
**Restructuring**	0.76	0.55	0.68	0.57	0.76	0.76

(Values in boldface add up to one; values in regular face are sub-criteria and also add up to one. The larger the value, the more important the decision criterion.) Numbers in parentheses are the number of stakeholders in each group.

### Data collection

Preferences for the IT innovations were elicited through an online survey. The online questionnaire included an AHP example to illustrate how the respondents were to score their preferences for the IT innovations, and the relative importance of the decision criteria. Subsequently, the decision criteria and IT innovations were described. As a first step of the preference elicitation, the respondents were asked to pairwise compare the importance of the criteria. To compare all criteria, the respondents judged seven pairwise comparisons on the validated AHP scale, ranging from equally important to extremely important. As a second step, the respondents compared, for each of the decision criteria, their preferences for the IT innovations in comparison with current care. The respondents judged 63 pairwise comparisons, to prioritize the nine IT innovations according to the seven decision criteria. This time the AHP scale ranged from equally preferred to extremely preferred. On the basis of these judgments, the overall priorities for the IT innovations were calculated with the AHP.

## Results

### Importance of the decision criteria

Table 2 shows the importance of the decision criteria to the stakeholder groups. The priorities of all main decision criteria (efficiency, health gain, satisfaction and investment) and all sub-criteria related to the same main criterion sum up to one. The scores are to be interpreted as relative weights of importance; the higher the score, the higher the relevance of the criterion according to the stakeholder group.

Within the stakeholder groups, the consistency ratios are 0.02 for patients, 0.04 for nurses, 0.13 for physicians, 0.10 for managers, 0.03 for health insurers, and 0.02 for policy makers. All consistency ratios are acceptable.

Efficiency is emphasized most strongly by policy makers (0.46; being the highest value), while patients, nurses and physicians least emphasize the importance of efficiency (0.22, 0.21 and 0.20 respectively). Health gain is judged to be most important by health insurers, nurses and patients (0.43, 0.35 and 0.42 respectively). Satisfaction is relatively important according to physicians, managers and nurses (0.33, 0.33 and 0.31 respectively). For managers and physicians, satisfaction (particularly patient satisfaction) can be even the most important decision criterion in selecting IT innovations.

### Preferences for the IT innovations

Table 3 shows the preferences of the different stakeholder groups for the IT innovations. A higher priority reflects a higher preference for this innovation. The priorities of all innovations add up to one. Results providing further insight into the distribution of priorities of individual respondents within each stakeholder group are presented as box plots in Additional file 3.

**Table 3:** Prioritization of innovations

**Innovation**	**Patients**	**Nurses**	**Physicians**	**Managers**	**Health insurers**	**Policy makers**
**Baseline innovation**	0.05	0.05	0.05	0.05	0.04	0.05
**Virtual consultation**	0.08	0.09	0.08	0.09	0.11	0.09
**Digital hospital portal**	0.10	0.12	0.11	0.12	0.12	0.11
**Planning operations**	0.12	0.13	0.11	0.11	0.09	0.11
**Telepathology**	0.08	0.07	0.09	0.08	0.10	0.07
**Barcodes**	0.16	0.13	0.09	0.12	0.10	0.13
**PDA**	0.09	0.10	0.16	0.09	0.11	0.09
**Telesurgery**	0.08	0.06	0.07	0.06	0.09	0.06
**Regional EPF**	0.15	0.15	0.19	0.14	0.11	0.11
**Self-tests**	0.09	0.10	0.06	0.13	0.13	0.18

Values in columns add up to one. The larger the value, the more important the innovation to the stakeholder group.

Patients appear to prefer medication barcodes and the regional electronic patient file (EPF), although their opinions on the value of the two innovations vary. Arguments are most strongly related to the perceived health gains and satisfaction resulting from the use of the two innovations.

Nurses also prefer most strongly the regional EPF and barcodes, together with planning software for operations. Similar to the patients, the nurses strongly differ in judgment about the expected value of barcodes and the EPF (see Additional file 3: Figure A2). The regional EPF is particularly valued because of its expected health gains and impact on satisfaction, the planning software because of the high satisfaction expected, and the barcodes because of the perceived health gains.

Physicians prefer the EPF most followed by the PDA because of their effects on satisfaction, health gains and efficiency. They particularly agree on the value of the PDA but less so on the value of the EPF.

Managers strongly prefer the regional EPF on account of its expected health gain and satisfaction, self-tests on account of the expected health gain, satisfaction and efficiency gain, and the digital hospital portal on account of the expected efficiency, satisfaction and health gain. No strong disagreements are found in the preference for the EPF, yet the value of self-tests is disputed among the managers.

Health care insurers prefer self-testing because of the expected efficiency and health gain, the digital hospital portal because of the low investment and efficiency gain, and the PDA because of its expected effect on satisfaction and health gain. Policy makers prefer self-testing on account of its expected increase in efficiency, barcodes on account of their health gains, and the digital hospital portal on account of the expected satisfaction to patients.

Policy makers strongly prefer self-tests, because of their strong, favorable effect on efficiency as expected by them. Self-tests are significantly favored over multiple other innovations (95% CI). This preference is followed by the preference for barcodes due to the expected health gain, and the digital hospital portal due to its expected effect on patient satisfaction.

Among the innovations, the regional EPF is in the top three preferences of four out of six stakeholders (patients, nurses, physicians and managers). However, the stakeholder most likely to bear a substantial part of the cost, the policy makers, did not appear to have much faith in this innovation at the time they filled out the questionnaire.

Even though preferences for barcodes strongly vary within most stakeholder groups, barcodes are among the top three preferences of four stakeholders (patients, nurses, physicians and policy makers). An important benefit of barcodes is perceived to be improved medication safety. Proper use will reduce incidents, and their resulting turmoil, (physical) damage and additional need for care. Policy makers, in particular, appear to be more unanimously convinced of this potential benefit.

A particularly large difference is found for digital hospital portals and self-tests. Managers, insurers and policy makers highly value these innovations, emphasizing their benefits, while the other stakeholder groups score these innovations lowly. The two innovations are clearly aimed at improving self-management and health care efficiency. These goals are important to the stakeholders who have to pay for the innovations (policy makers and insurers) and seem less important to the stakeholders who need to change their work practices because of increased self-management. Policy makers assigned a significantly higher priority to self-tests than patients did (95% CI).

## Conclusions and discussion

The main questions of this paper were: Do different stakeholders have different preference structures concerning the relative relevance of IT-innovations in health care? And, can these differences be understood from the differences in the costs and benefits associated with the innovations for the stakeholders? Understanding the differences in preferences and subsequent priorities in innovation agendas of different stakeholders may lead to an understanding of the differences in the speed of diffusion of innovations.

We found differences in preferences between six stakeholder groups with regard to nine IT innovations in hospitals. For example, substantial differences were found in the preference for self-testing. Patients who would play a vital role in the effective implementation of self-tests do not give self-tests a high priority. This can be partly understood by cost–benefit considerations. To conduct self-tests, patients have to learn new skills and take responsibility for their care process, which they may not aspire to do so. The benefits of more efficient health care are mainly found within the system.

The highest level of consensus between the six stakeholder groups was found for a regional EPF. Even the stakeholders who may have to bear substantial costs in terms of having to change their daily routines (i.e., nurses and physicians) see the added benefits of the innovation. The implementation of an EPF involves different costs for different stakeholders. Nurses and physicians need to change their work practices in the sense that many of their treatment-related activities are computerized. (This change depends on the interface, but working with stations, the change can be quite cumbersome.) Hospitals need to invest in IT systems and national policy makers need to invest in regional data infrastructures. The perceived benefits of EPF are, however, not without controversy. Among the stakeholder groups, judgments differed most strongly on the EPF as well as medicine barcodes and self-tests.

The strongest preferences found in this study are those of the policy makers for self-tests, and those of the physicians for PDAs. For the policy makers, self-tests could result in a decrease in costs because part of the disease diagnosing process is done by the patients themselves, thereby reducing the number of general-practitioner consultations. Therefore, they expect a gain in efficiency of care. The physicians prefer the PDA, probably because of the ease of use, with all relevant data being readily available at the patient’s bedside or the operating table. For the PDA to work, a well-functioning EPF is necessary, which is their second preference. This seems to indicate that the physicians in our study will embrace the benefits of a well-functioning EPF with a mobile interface.

There were indications that stakeholder preferences can be understood from the benefits they gain from the innovation. We also saw that these benefits differ between stakeholders, affecting their prioritization of which innovation to implement first. We saw little direct evidence of costs having a major effect on the decisions of the stakeholders.

In terms of the relative importance of the decision criteria, policy makers deviate from the other stakeholders. To policy makers, efficiency in health care is the most important factor in deciding whether an innovation has added benefits. For patients, nurses and insurers, health gains constitute the decisive factor in deciding on beneficial innovations, and for physicians and managers, the decisive factor is (patient) satisfaction.

All policy makers operated on the national level and were very concerned about the increasing health care expenditure. This concern was translated in the high priority of the efficiency gains due to the innovations. In the interviews, policy makers indicated that they use different tools to steer the innovation process. Besides implementing taxes, making inspections and providing funding, they may establish contact between innovators and care providers to start meaningful cooperation. The interviews revealed an important difference between policy makers and other stakeholders, in that the majority of them found it difficult, if not impossible, to appreciate the implications of single innovations. They were better equipped to discuss general directions and macro consequences of innovations.

None of the stakeholders indicated that they consider investment to be the most relevant factor in deciding on the added value of an innovation. It should be noted that these outcomes are based on subjective expectations, and not on evidence from health economic studies. A drawback of this study is that the respondents reported their subjective expectations and these expectations may differ from real life choices that stakeholder groups make. The relatively small weight that was assigned to the costs may point towards such bias.

We measured the preferences of the various stakeholders. These preferences are likely to affect the decisions on adopting innovations. The preferences appeared to be related to the cost–benefit ratio of each stakeholder. This cost–benefit ratio is affected by the stakeholders’ position in an organization or a health care system. We did not study the actual effect of preferences on decision making. The effect of preferences on decisions about adoption may be the topic of another study.

Even though we tried to be specific in our descriptions of innovations, we also had to find a description that would be understood by all stakeholders. The result may have been too general a description of the innovations. For instance, the EPF has many different applications and methods of operation. It may have been that different respondents had different ideas of the EPF while answering the question, based on their own experience.

Another way to improve this study is to increase the number of respondents. It was found particularly difficult to have physicians answer the questionnaire. We received feedback that some of them found it too difficult to answer the questions, or that completing the questionnaire was too time consuming. Still, we explored the possibilities for the use of AHP in innovation research, and even with the relatively small number of respondents, believe that we have obtained promising results that can be understood in terms of the stakeholder framework. Later studies may build on the ideas presented in this paper.

If in subsequent studies it is possible to increase the number of respondents, further differentiations among a larger number of stakeholders may be possible. For instance, we did not further differentiate the management of hospitals. However, when managers operate on different levels or in different positions in an organization, this may affect their preferences. The differences are expected to be smaller than those between the stakeholder groups we included, but these small differences may delay the innovation processes. With further differentiation, an even more diverse picture of differences between stakeholders is likely to emerge. An increased number of respondents may also be used to correct analyses for aspects such as cultural differences and resemble the wider variety of health care organizations.

What would we expect concerning the speed of the diffusion of the innovations that were included in this study? Virtual consultations, telepathology and telesurgery did not appear in any of the top three preferences of any of the stakeholders. We therefore do not expect rapid and broad diffusion of these innovations soon. The planning of operations is ranked by patients and nurses to be important; however, it will need to be implemented by the hospital organization. We believe that the planning is an inevitable step in creating an efficient hospital and expect it to diffuse at a medium rate. Self-tests are highly ranked by the stakeholders that do not use it (managers, health insurers and policy makers), but not by those who will need to use it. We therefore expect a slow implementation of self-tests. For the digital hospital portal, we find a similar preference structure. However, these kinds of implementations are performed more by the hospital organization. This type of innovation may therefore diffuse more quickly. Barcodes are seen as useful innovations by most of the stakeholders. The use of barcodes may therefore diffuse quickly with the support of all stakeholders. The use of a PDA is mainly found to be important by the physicians and the EPF by most of the stakeholders close to the care process. Even though the two are seen in this study as separate innovations, the PDA can be used as a mobile interface for the EPF. If these two technologies are combined, a mobile source of information becomes available to the care professionals, possibly integrating data input more closely to the care process. The combination of readily available information with quick and easy data input may create more momentum for the ongoing diffusion of registration of patient data. Future research may empirically test these expectations.

## Conclusion

For innovation research, this means that when studying implementation, taking account of the number of stakeholders involved in the implementation decision and how they benefit from or pay for the implementation and use of the innovation may increase the understanding of the (lack of) speed of the diffusion process. If one of the stakeholders in any part of the process faces costs that exceed the benefits, the process may be stopped in the implementation phase where involvement of the particular stakeholder is crucial. Knowing more about costs and benefits at a stakeholder level may therefore better explain why diffusion of particular innovations is delayed than taking into account possible costs and benefits on a more aggregate level.

## Competing interests

The authors have no competing interests as described in the authors’ instructions for this journal, nor other competing interests.

## Authors’ contributions

ML developed the idea of the paper and the theoretical framework. He also recruited respondents, conducted and interpreted interviews, judged the results of the literature searches, and wrote the text. MH conducted a literature search, made the online questionnaire, conducted AHP analyses and wrote the text. All authors read and approved the final manuscript.

## Pre-publication history

The pre-publication history for this paper can be accessed here:

http://www.biomedcentral.com/1472-6947/13/91/prepub

## Supplementary Material

Additional file 1 Search queries of literature search. Click here for file

Additional file 2 Table with description, expected added value and literature of innovations that were included in the AHP. Click here for file

Additional file 3 Box plots of stakeholder preferences of nine innovations. Click here for file
