# Supplementary material for: Differentiating innovation priorities among stakeholder in hospital care
Source: BMC Med Inform Decis Mak. 2013 Aug 16;13:91. doi: 10.1186/1472-6947-13-91 (PMC3751765; doi:10.1186/1472-6947-13-91)
Supplement: Additional file 1 — Search queries of literature search. [file 1472-6947-13-91-S1.docx]

**Additional file 1 Search queries**

**Web of Science: ICT**

(TS = (("new techn*" OR "new product*" OR "emerging techn*" OR innovation*) AND (IT OR ICT OR "information technolog*" OR "communication techn*") AND (health OR patient* OR clinic* OR medic*) AND (efficiency))) AND Language=(English) AND Document Types=(Article)

Refined by: [excluding] Web of Science Categories=( ENVIRONMENTAL STUDIES )

Timespan=2005-2011. Databases=SCI-EXPANDED, SSCI, A&HCI, CPCI-S, CPCI-SSH.

**Scopus**

("new techn*" OR "new product*" OR "emerging techn*" OR innovation*) AND (IT OR ICT OR "information technolog*" OR "communication techn*") AND (efficiency) AND (hasabstract[text] AND English[lang] AND "last 5 years"[PDat])
