# Supplementary material for: Differentiating innovation priorities among stakeholder in hospital care
Source: BMC Med Inform Decis Mak. 2013 Aug 16;13:91. doi: 10.1186/1472-6947-13-91 (PMC3751765; doi:10.1186/1472-6947-13-91)
Supplement: Additional file 2 — Table with description, expected added value and literature of innovations that were included in the AHP. [file 1472-6947-13-91-S2.docx]

**Additional file 2 Table with description, expected added value and literature of innovations that were included in the AHP**

Innovations are practices or objects that are perceived as new by the actor who adopts them. It is also suggested that innovations improve upon the status quo.

The table gives a description of the innovations included in the AHP and their expected benefits. It is inherent in studying innovations that only some time after the introduction of an innovation the full scale of its benefits and (hidden) problems become apparent. Hence, the expected benefits are judgments based on incomplete information and the expectation may change over time.

| **Innovation** | Description and additional literature | Expected benefits |
| --- | --- | --- |
| Regional EMR | A regional EMR is an IT application that enables the exchange of patient information between health care providers in a region. The information of patients who receive care from multiple providers (simultaneously or consecutively) is accessible for all providers involved.  Mills, TR, Vavroch, J, Bahensky, JA, Ward, MM. **Electronic medical record systems in critical access hospitals: leadership perspectives on anticipated and realized benefits**. *Perspectives in health information management / AHIMA, American Health Information Management Association*, 2010, 7. | Faster and more accurate information exchange between health care providers, higher quality of care, less redundancy, increased patient safety. |
| Computer-assisted telesurgery using a robot | A surgery robot (mechanical arm that can be equipped with surgical tools), with remote control and monitors, which serves as a tool used by surgeons during surgery, possibly over a large geographic distance.  Masic, I, Pandza, H, Kulasin, I, Masic, Z, Valjevac, S. **Tele-education as method of medical education.** *Medicinski arhiv*, 2009, 63 (6), pp. 350–353.  Citak, M, Haasper, C, Kendoff, D, Geerling, J, Ortega, G., Krettek, C, Kfuri, M., Hüfner, T. **Preliminary clinical experience using a newly developed minimal-invasive reference base in computer assisted foot surgery.** *Technology and Health Care* 2006, 14 (6), pp. 515–519. | Less pain, shorter recovery period, smaller scars for patient.  Cost–benefit ratio of the technique is not undisputed. |
| Barcode medication administration technology | Medication stored and administered in health care institutions is registered with labels having barcodes. The staff uses (hand-held) registration devices to monitor the flow of medication. This is registered and used as input for new ordering and monitoring to improve medication safety.  Carayon, P, Wetterneck, TB, Hundt, AS, Ozkaynak, M, DeSilvey, J, Ludwig, B, Ram, P, Rough, SS. **Evaluation of nurse interaction with bar code medication administration technology in the work environment.** *Journal of Patient Safety* 2007, 3 (1), pp. 34–42. | Increased patient safety through the easier tracking of medication and materials, less waste because of lost products or expired medication, less storage required. |
| Telepathology | Telepathology is the practice of diagnostic pathology performed at a distance, with images viewed on a video monitor rather than directly through a (light) microscope.  Weinstein RS, Bhattacharyya AK, Graham AR, Davis JR. **Telepathology: a ten-year progress report.** *Human Pathology,* 1997;28:1-7. 2  Weinstein RS. Prospects Weinstein, R.S., Graham, A.R., Richter, L.C., Barker, G.P., Krupinski, E.A., Lopez, A.M., Erps, K.A., Bhattacharyya, A.K., Yagi, Y., Gilbertson, J.R.  **Overview of telepathology, virtual microscopy, and whole slide imaging: prospects for the future**. *Human Pathology*, 2009, 40 (8), pp. 1057-1069. | Use of telecommunication (video and/or microscope) enables quick diagnosis in some surgical procedures over a distance, pathologist does not need to be present in the hospital to give diagnosis, reduces travel costs, highly specialized knowledge is made accessible to peripheral services. |
| Self-testing and online automated management | Self-testing and on-line automated management consists of devices (possibly on line) that enable patients (with chronic diseases) to transmit relevant biomedical information to a care provider. In some cases, the devices enable registration and transmission of, for example, blood values without intervention of the patient, whole in other cases, the patient has to enter the data manually on a website.  Bussey, HI (2011) **Transforming oral anticoagulation by combining international normalized ratio (INR) self testing and online automated management.** *Journal of Thrombosis and Thrombolysis, 2011,* 31 (3) special issue, pp. 265–274. | Reduction of (travel) time and expenses for patients (with chronic diseases), increased monitoring options, increased options to decrease time spent in hospital. |
| Digital hospital portal | Digital hospital portals are (parts of) websites of hospitals that enable patients to access their patient files, make and/or reschedule appointments and re-order medication. They may also enable peer-to-peer or peer-to-physician communication or may facilitate the transmission of other forms of information for patients.  Weingart, SN, Rind, D., Tofias, Z., Sands, DZ. **Who Uses the Patient Internet Portal? The PatientSite Experience.** *Journal of the American Medical Informatics Association, 2006*, 13 (1); pp. 91–95. | Improvement of communication between patient and hospital, appointments can be made and changed on line,  facilitates patient–specialist consultation, facilitates patient–patient communication. |
| Virtual consultations | Virtual consultations consist of two-way video conferencing between two clinicians. Different types of specialists can discuss patients over a large distance.  Schwamm, LH, Rosenthal, ES, Hirshberg, A, Schaefer, PW, Little, EA, Kvedar, JC, Petkovska, I, Koroshetz, WJ, Levine, SR  **Virtual TeleStroke Support for the Emergency Department Evaluation of Acute Stroke.** *Acad Emerg Med November,* 2004, 11 (11), pp. 1193–1197 | Reduction of travel time and costs, specialist care becomes more widely available. |
| Software to support operating room planning | Software to plan operations in a hospital and thus optimize the use of operation room facilities.  Testi, A; Tanfani, E **Tactical and operational decisions for operating room planning: Efficiency and welfare implications**  *Health Care Management Science* , 2009, 12(4), pp. 363–373 DOI: 10.1007/s10729-008-9093-4 2009 | More efficient use of operation facilities. |
| PDA with decision support | Hardware such as tablets and smart phones may be used to interface with computer systems (e.g., the electronic medical record system) of a health care institution. Using a PDA enables the health care professional to walk freely in the organization and enter and request patient data at the patient’s bedside. When used for decision support, changes in medication are monitored directly for interacting medications.  Gill PS, Kamath A, Gill TS, **Distraction: an assessment of smartphone usage in health care work settings**. *Risk Management and Healthcare Policy* 2012, 2012(5):105–114 | Increased user friendliness of the interface with the ICT containing patient information. |
