# Supplementary material for: Differentiating innovation priorities among stakeholder in hospital care
Source: BMC Med Inform Decis Mak. 2013 Aug 16;13:91. doi: 10.1186/1472-6947-13-91 (PMC3751765; doi:10.1186/1472-6947-13-91)

**Additional file 3** **Box plots**

Figure A1. Box plots of priorities of the IT innovations as assigned by patients. The horizontal line in a box represents the median priority, and the circle and star the outliers. The bottom and top of each box show the 25th and 75th percentiles, and the vertical lines extending from the boxes the 95% confidence interval.


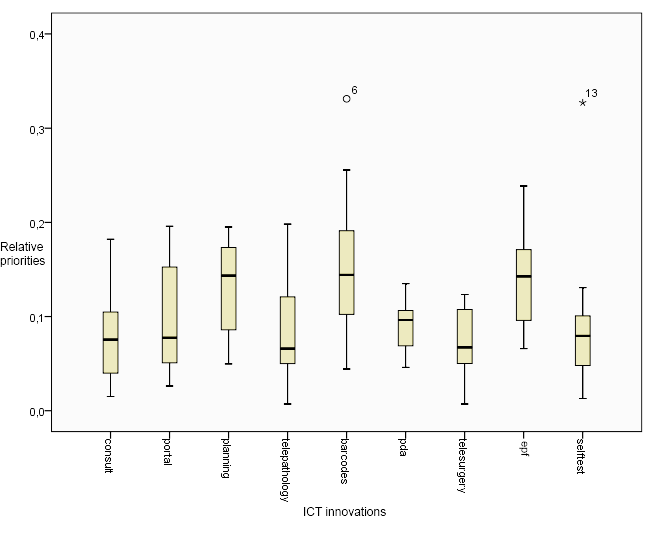


Figure A2. Box plots of priorities of the IT innovations as assigned by nurses. The horizontal line in a box represents the median priority, and the circles and star the outliers. The bottom and top of each box show the 25th and 75th percentiles, and the vertical lines extending from the boxes the 95% confidence interval.


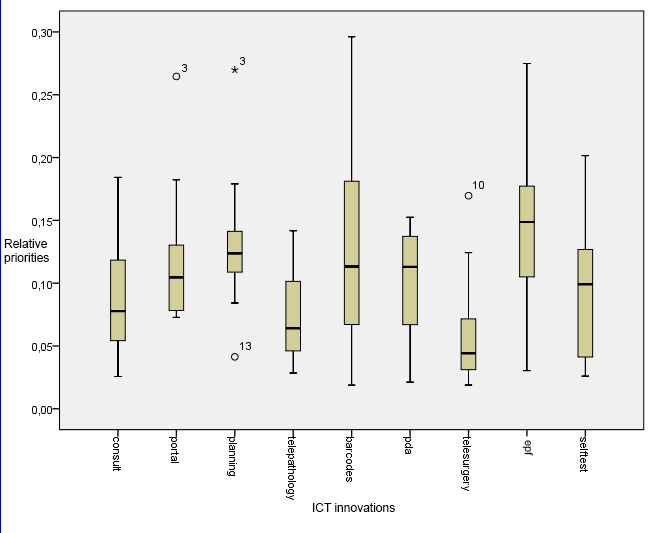


Figure A3. Box plots of priorities of the IT innovations as assigned by physicians. The horizontal line in a box represents the median priority, and the circles the outliers. The bottom and top of each box show the 25th and 75th percentiles, and the vertical lines extending from the boxes the 95% confidence interval.


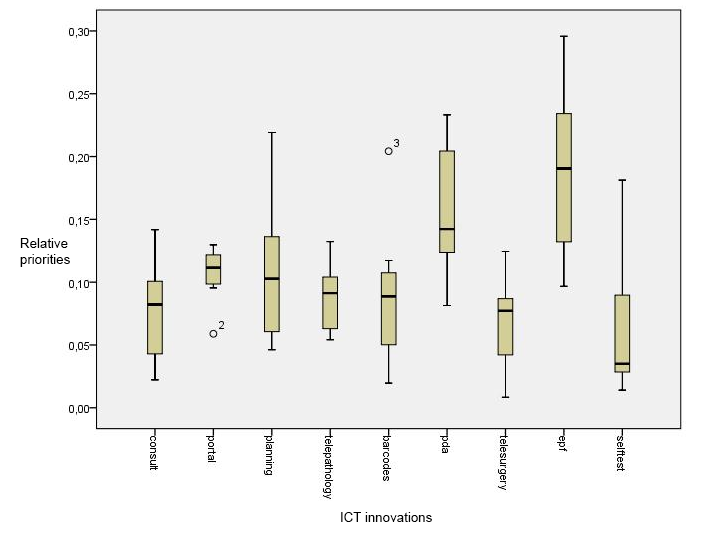


Figure A4. Box plots of priorities of the IT innovations as assigned by managers. The horizontal lines in a box represents the median priority, and the circles the outliers. The bottom and top of each box show the 25th and 75th percentiles, and the vertical lines extending from the boxes the 95% confidence interval.


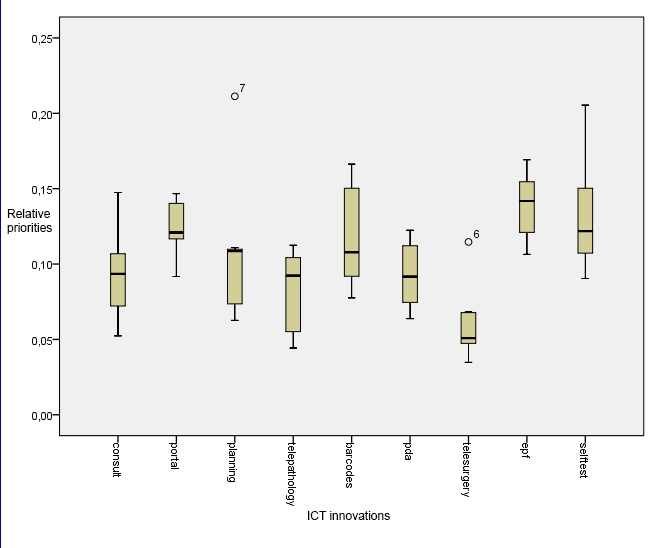


Figure A5. Box plots of priorities of the IT innovations as assigned by health insurers. The horizontal line in a box represents the median priority. The bottom and top of each box show the 25th and 75th percentiles, and the vertical lines extending from the boxes the 95% confidence interval.
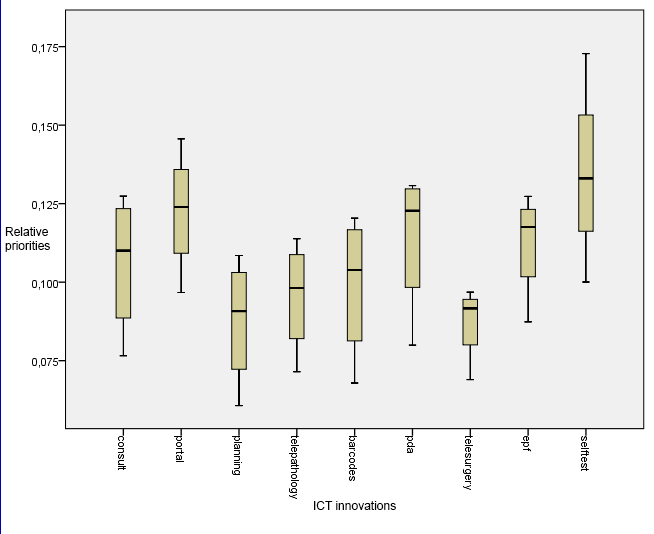


Figure A6. Box plots of priorities of the IT innovations as assigned by policy makers. The horizontal line in a box represents the median priority, and the circles and stars the outliers. The bottom and top of each box show the 25th and 75th percentiles, and the vertical lines extending from the boxes the 95% confidence interval.


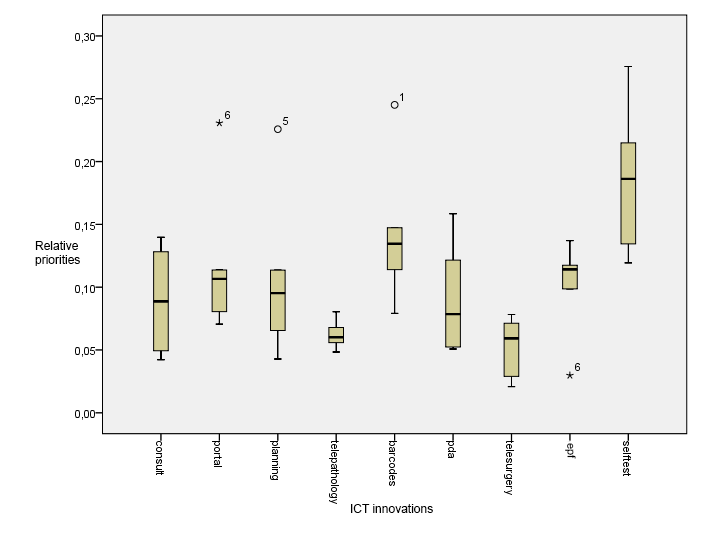

Supplement: Additional file 3 — Box plots of stakeholder preferences of nine innovations. [file 1472-6947-13-91-S3.docx]
